# Supplementary material for: Evaluation of Spliceosome Protein SmD2 as a Potential Target for Cancer Therapy
Source: Int J Mol Sci. 2024 Dec 6;25(23):13131. doi: 10.3390/ijms252313131 (PMC11642717; doi:10.3390/ijms252313131)
Supplement: Supplementary file 1 [file ijms-25-13131-s001.zip › Supplemental Figures.pdf]

Supplemental figures

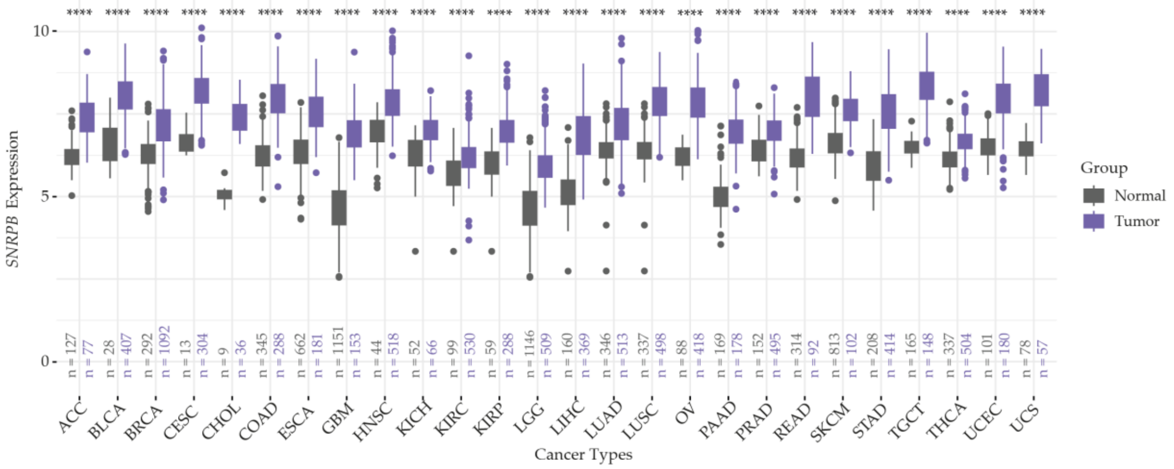

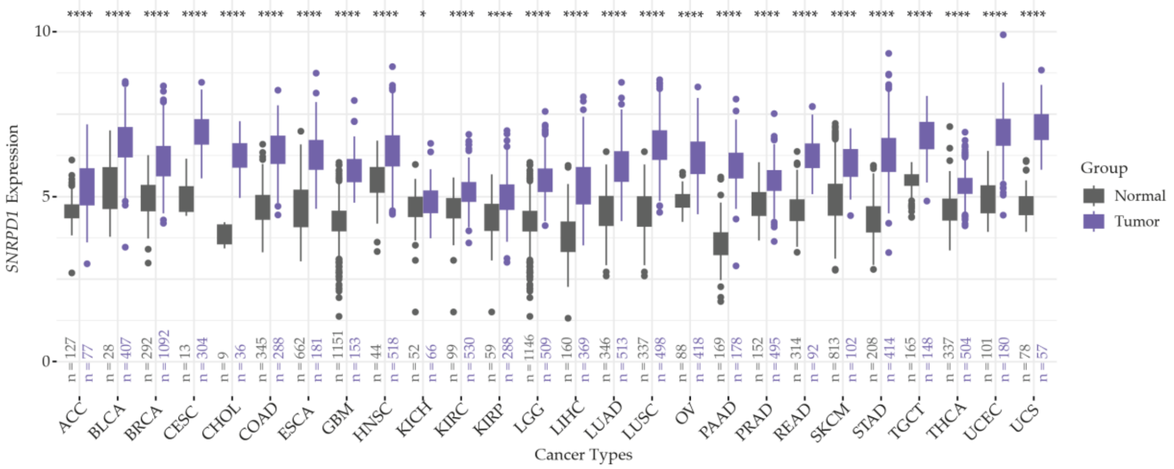

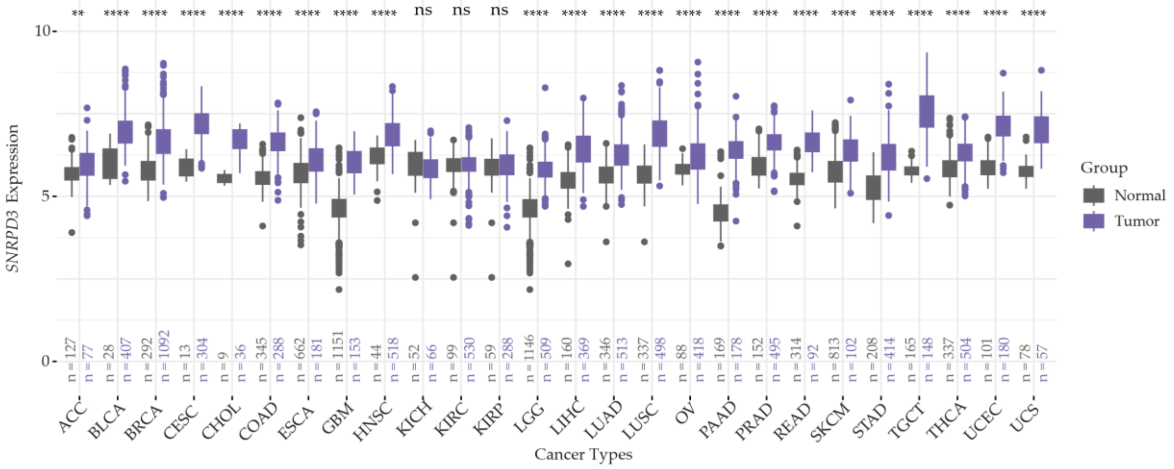

(Continued on the next page)

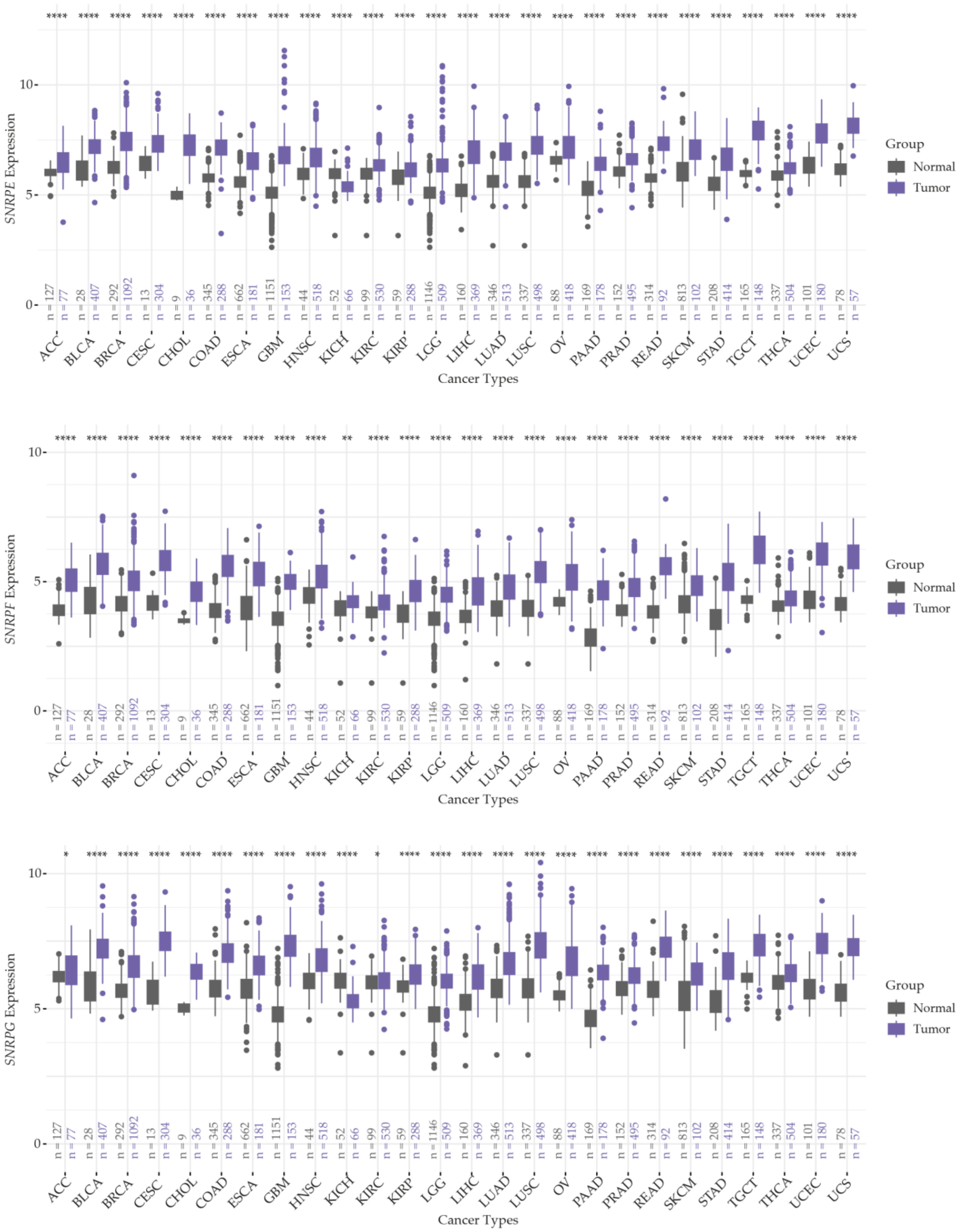

Figure S1. *SNRPB*, *SNRPD1*, *SNRPD3*, *SNRPE*, *SNRPF* and *SNRPG* mRNA expression comparisons in 26 solid cancer types and their matched normal tissues. The gene expression data was retrieved from TCGA Pan-Cancer and GTEx datasets (units: log2(TPM+0.001)). The sample size for each group in each comparison is marked by “n”. Differences between normal tissues and tumor tissues were tested by unpaired Student’s *t*-test (ns, not significant; *, $p < 0.05$; **, $p < 0.01$; ****, $p < 0.0001$).

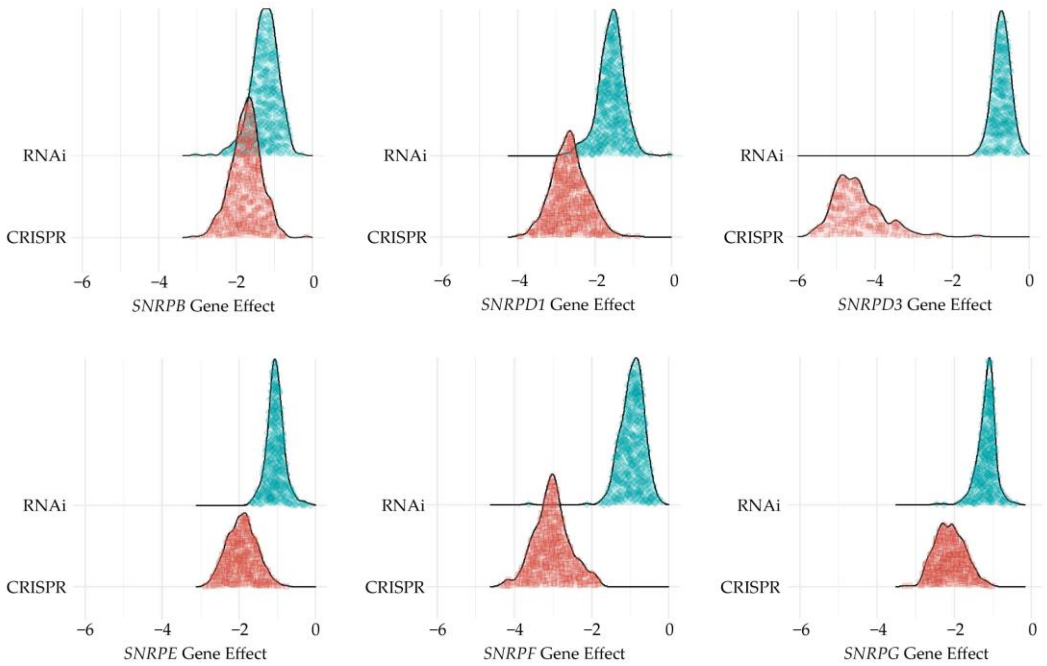

Figure S2. RNAi and CRISPR dependency scores of the other 6 Sm genes (*SNRPB*, *SNRPD1*, *SNRPD3*, *SNRPE*, *SNRPF* and *SNRPG*). RNAi and CRISPR dependency scores were retrieved from the Depmap RNAi (Achilles+DRIVE+Marcotte, DEMETER2) dataset and the CRISPR (DepMap 23Q4 Public+Score, Chronos) dataset. The collected data included 346 human cancer cell lines from RNAi screens and 469 human cancer cell lines from CRISPR screens, from a variety of tissue origins. A score of 0 is equivalent to a gene that is not essential; a score of -1 corresponds to the median of all common essential genes. The lowest score indicates the most sensitive cells.

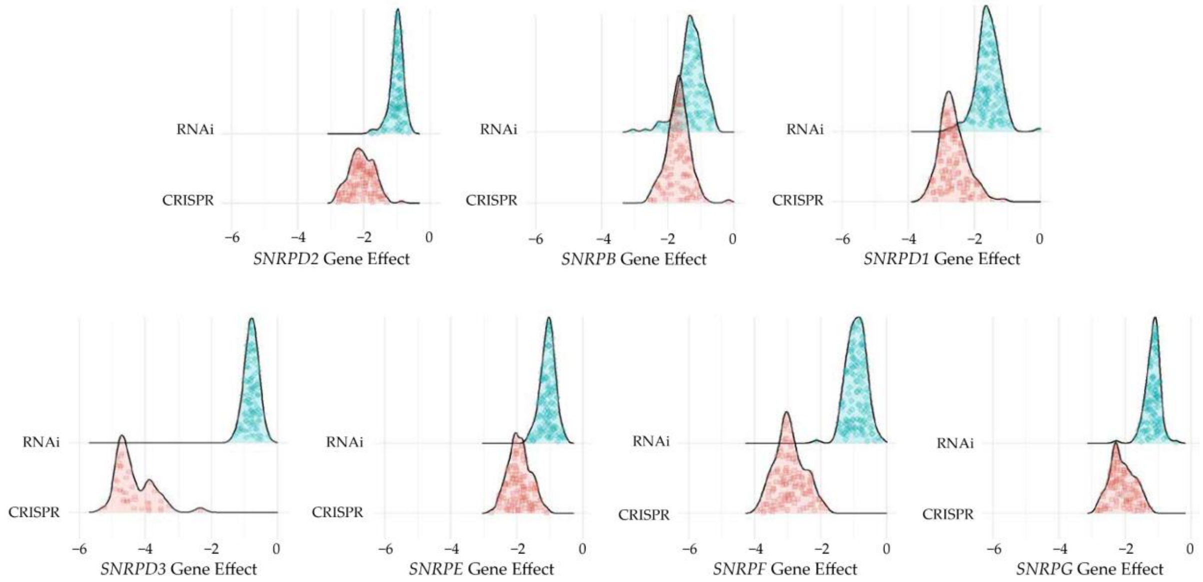

Figure S3. RNAi and CRISPR dependency scores of the 7 Sm genes (*SNRPD2*, *SNRPB*, *SNRPD1*, *SNRPD3*, *SNRPE*, *SNRPF* and *SNRPG*) for NSCLC only. RNAi and CRISPR dependency scores were retrieved from the Depmap RNAi (Achilles+DRIVE+Marcotte, DEMETER2) dataset and the CRISPR (DepMap 23Q4 Public+Score, Chronos) dataset. The collected data included 113 human NSCLC cell lines from RNAi screens and 119 human NSCLC cell lines from CRISPR screens. A score of 0 is equivalent to a gene that is not essential; a score of -1 corresponds to the median of all common essential genes. The lowest score indicates the most sensitive cells.

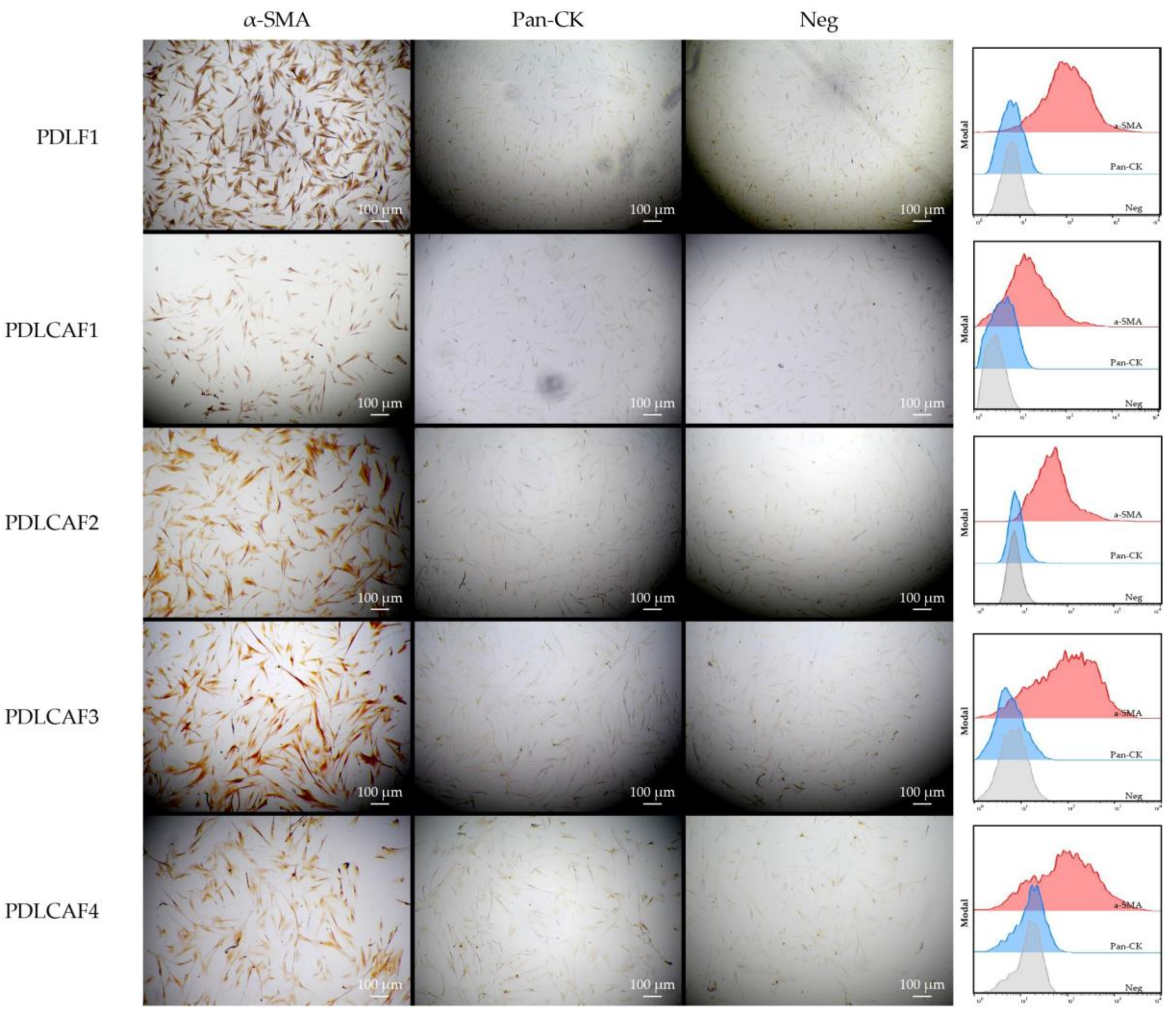

Figure S4. Characterization of patient-derived cells cultured from lung tumor resections. Immunocytochemistry bright-field microscopy images showing the expression of α-SMA and Pan-Cytokeratin. Flow cytometry histogram illustrating the distribution of α-SMA and Pan-Cytokeratin expression across the cell populations. All cell cultures were concluded to consist mainly or exclusively of α-SMA-positive (myo)fibroblasts. PDLF1 was grown from a resected solitary fibrous tumor; PDLCAF1-4 from resected NSCLC tumors.

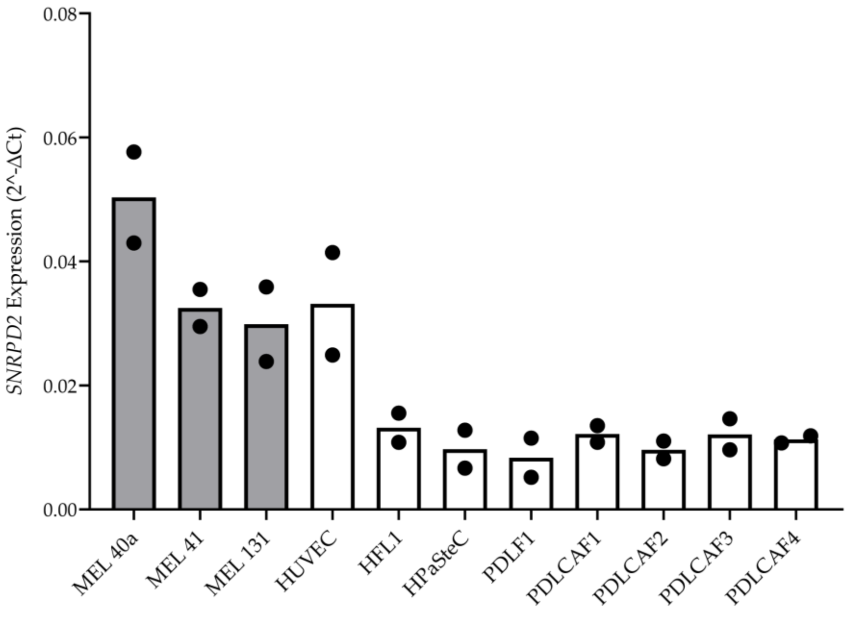

Figure S5. Endogenous *SNRPD2* expression levels in human cell cultures as determined by RT-qPCR analysis. *SNRPD2* expression was normalized by *ACTB* expression. The data were from two independent experiments performed in triplicate.

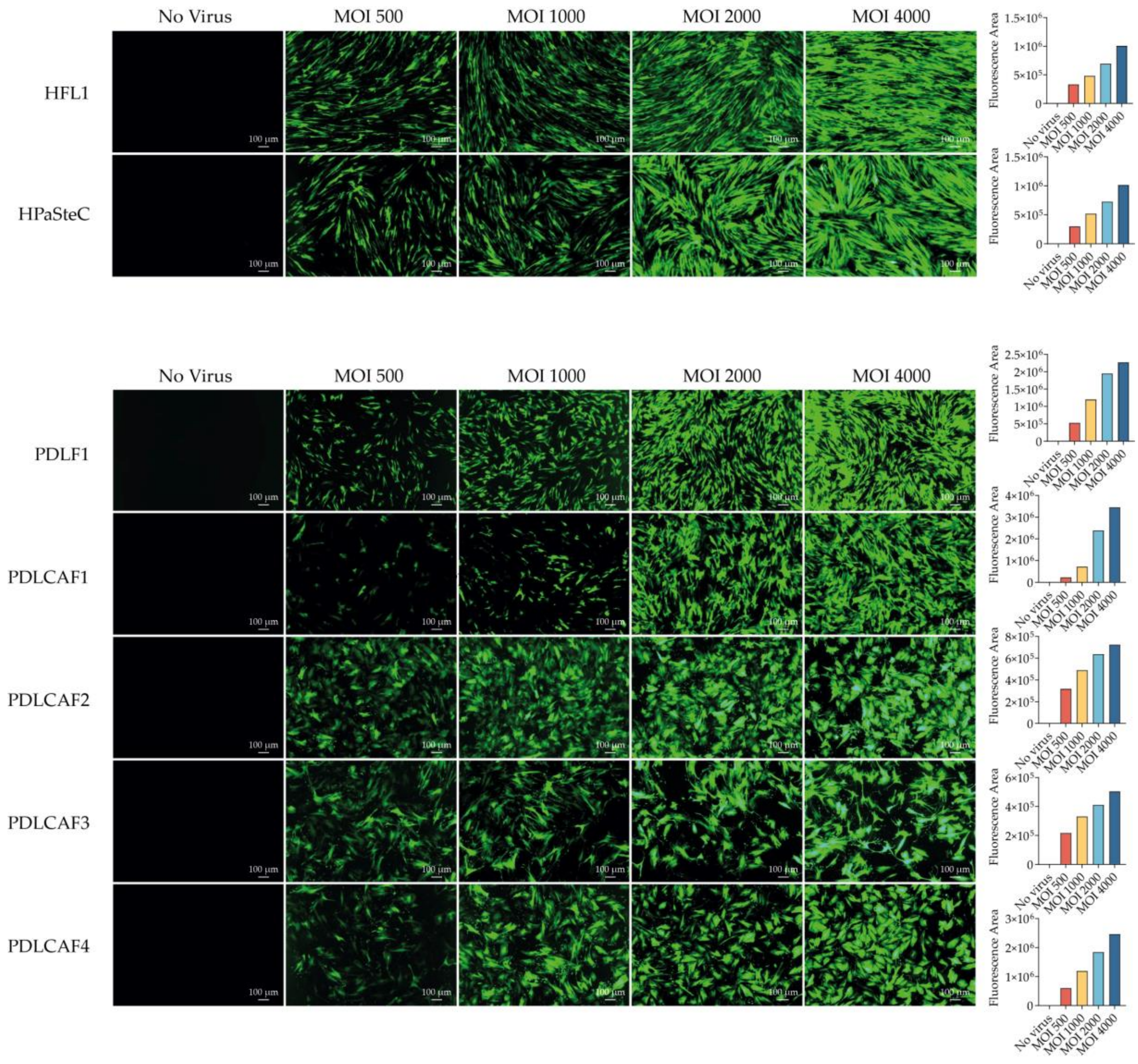

Figure S6. Transduction of non-malignant cell cultures with LV-eGFP at varying MOIs. Fluorescence microscopy images were taken three days post-transduction. Quantification of green fluorescent areas in the field of view was done using ImageJ software. A dose-dependent increase in transduction was observed on all cultures. A multiplicity of infection 4000 gc/cell was saturating.

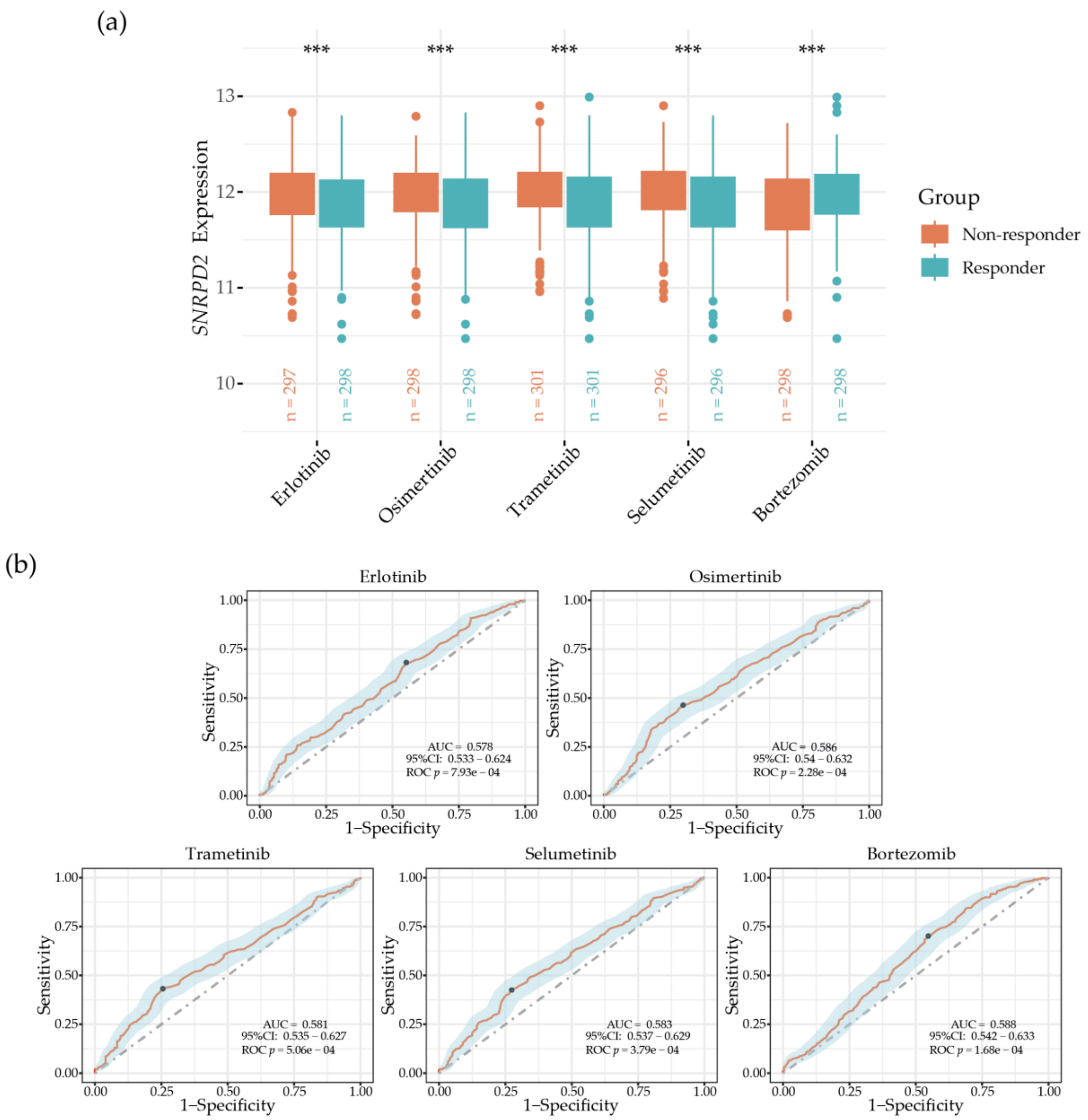

Figure S7. Association between *SNRPD2* expression and response of cancer cells to treatment with anti-tumor drugs in vitro**.** Box-plots (**a**) and ROC curves (**b**) of *SNRPD2* expression and response to FDA-approved anti-tumor drugs. The *SNRPD2* expression data (RMA normalized Affymetrix Human Genome array) for cancer cell lines was downloaded from the GDSC1000 resource. CellTiterGlo assay was used for cell viability and AUC evaluation, and AUC values were extracted from the GDSC2 database. *SNRPD2* expressions in non-responder and responder groups were compared using the Mann–Whitney U test (**a**) and ROC test (**b**). ***, $p < 0.001$; the strongest cut-off is shown as a black dot, and the 95% confidence interval for the AUC is shown as a light blue ribbon on the ROC curve. The figure includes the selected drugs for which a significant association between *SNRPD2* expression and sensitivity was observed, but that did not meet the AUC > 0.6 criterion.

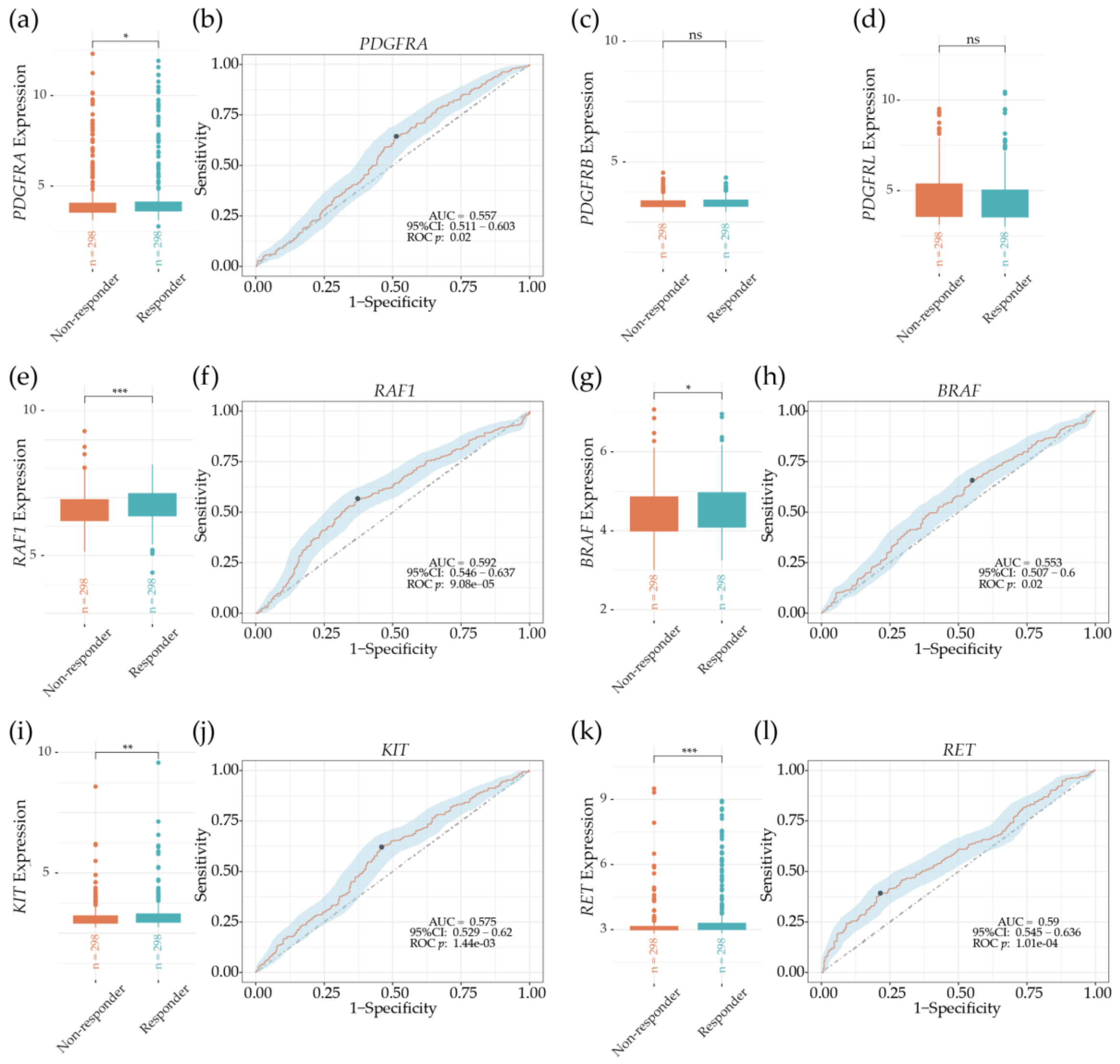

Figure S8. Box-plots and ROC curves of response to sorafenib and expression of known sorafenib targets. The gene (*PDGFRA*, *PDGFRB*, *PDGFRL*, *RAF1*, *BRAF*, *KIT*, *RET*) expression data (RMA normalized Affymetrix Human Genome array) for cancer cell lines was downloaded from the GDSC1000 resource. CellTiterGlo assay was used for cell viability and AUC evaluation, and AUC values were extracted from the GDSC2 database. Individual gene expressions in non-responder and responder groups were compared using the Mann–Whitney U test (**a**,**c**,**d**,**e**,**g**,**i**,**k**) and ROC test (**b**,**f**,**h**,**j**,**l**). ns, not significant; *, $p < 0.05$; **, $p < 0.01$; ***, $p < 0.001$; the strongest cut-off is shown as a black dot, and the 95% confidence interval for the AUC is shown as a light blue ribbon on the ROC curve.
